# The impact of vagus nerve stimulation on the most disabling seizures: A retrospective study in adults with drug-resistant epilepsy

**DOI:** 10.1007/s10072-026-08868-x

**Published:** 2026-02-10

**Authors:** Anna Scarabello, Luca Zanuttini, Martino Schettino, Lorenzo Muccioli, Lorenzo Ferri, Francesca Bisulli, Ilaria Naldi, Lilia Volpi, Roberto Michelucci, Antonella Boni, Valentina Gentile, Mino Zucchelli, Paolo Tinuper, Matteo Martinoni, Barbara Mostacci

**Affiliations:** 1 Department of Biomedical and Neuromotor Sciences (DIBINEM), University of Bologna, Bologna, Italy; 2 Statistics and Epidemiology Department, IRCCS Istituto delle Scienze Neurologiche di Bologna, Bologna, Italy; 3 Epilepsy Program, Full Member of European Reference Network EpiCARE, IRCCS Istituto delle Scienze Neurologiche di Bologna, Pad G1 - Ospedale Bellaria, Via Altura 3, 40139 Bologna, Italy; 4 Neurology Department, AUSL Azienda Unità Sanitaria Locale di Imola, Imola, Italy; 5 Neurology Department, IRCCS Istituto delle Scienze Neurologiche di Bologna, Bologna, Italy; 6 Paediatric Neuropsychiatry department, IRCCS Istituto delle Scienze Neurologiche di Bologna, Bologna, Italy; 7 Neurosurgery Department, IRCCS Istituto delle Scienze Neurologiche di Bologna, Bologna, Italy; 8 University of Bologna, Bologna, Italy

**Keywords:** Epilepsy surgery, Neuromodulation, VNS, DRE, Tonic-clonic seizures

## Abstract

**Purpose:**

To evaluate the long-term efficacy of vagus nerve stimulation (VNS) in reducing tonic-clonic seizures (TCS), drop attacks, and seizure clusters in adults with drug-resistant epilepsy (DRE).

**Methods:**

This retrospective, single-center study included adults with DRE who received VNS and had ≥ 12 months of follow-up. Data were collected pre-implantation (T0), at 12 months (T1), and last follow-up (T2). Outcomes included reduction in total seizure frequency and severity, frequency of TCS and drop attacks, and frequency/duration of seizure clusters. Battery replacement and tolerability were also assessed.

**Results:**

Eighty-seven subjects (51 males, median age 33 at T0) with a mean follow-up of 8 years were analyzed. At T2, 54% showed reduced total seizure frequency and 71% reported decreased severity/duration. Among those with baseline TCS, 75% (T1)–80.6% (T2) had frequency reduction, with 20.6% seizure-free at T2. Drop attacks improved in > 70% of cases, with resolution in 21.8% at T2. Over 80% with seizure clusters reported decreased frequency/duration at both T1 and T2. Response rates for specific seizure types significantly exceeded non-response (*p* < 0.001). Battery replacement was required in 85.7% near depletion. Side effects occurred in 49.4%, mostly mild/transient; major complications were rare.

**Conclusion:**

VNS exerts a robust effect on the most disabling seizures—TCS, drop attacks, and seizure clusters—surpassing its impact on overall seizure burden. Benefits appear within the first year and persist long term. These findings support a more tailored approach to VNS candidate selection, advocating earlier use in patients with refractory, harmful seizures not eligible for curative surgery.

## Highlights

- VNS efficacy should be assessed not only by overall seizure reduction, but by its impact on the most disabling seizure types that affect daily functioning
- VNS reduces tonic-clonic seizures in up to 80% of adults with drug-resistant epilepsy
- Over 20% of patients achieved complete freedom from drop attacks after VNS
- VNS cuts seizure clusters frequency or duration in more than 80% of cases.

## Introduction

 Vagus nerve stimulation (VNS) was introduced in Europe in 1994 [1] as an adjunctive treatment for people with drug-resistant epilepsy (DRE) who are either ineligible or unresponsive to curative surgery [2, 3]. Initially approved for focal seizures in children over 12 years, the EMA indication now encompasses medically refractory focal and generalized epilepsies across all age groups [4]. The effectiveness of VNS typically becomes evident after 6–12 months of treatment and has been shown to improve over time [5–7]. Long-term follow-up data indicate that approximately 45–65% of patients experience a >50% reduction in seizure frequency [4]. Several clinical variables have been explored as potential predictors of VNS response to improve candidate selection for this treatment. Early implantation and shorter epilepsy duration prior to intervention are both associated with better outcomes [4, 6]. While children generally exhibit a more favorable response compared to adults, no significant age-related differences have been observed within adult populations [4]. Non-structural etiologies were typically associated with better outcomes compared to structural cases [4]. Nonetheless, favorable responses have also been documented in specific structural conditions, such as post-traumatic epilepsy [8] and tuberous sclerosis [4]. Although several clinical features have been associated with more favorable outcomes following VNS, no consistent or universally reliable predictor of response has been validated to date [4, 9]. Within the literature examining VNS efficacy across specific seizure-types, a relatively unexplored aspect is its impact on seizures that most significantly affect morbidity, mortality, and daily functioning — namely, tonic-clonic seizures (TCS) and seizures that may lead to falls [6, 10, 11]. Furthermore, although acute VNS implantation has shown potential efficacy in treating super-refractory status epilepticus [12], and magnet use can interrupt seizure clusters [13], evidence regarding the efficacy of chronic VNS neuromodulatory therapy in mitigating seizure clusters remains scarce.

This study evaluates the long-term outcomes of VNS, focusing on the seizure types we considered most disabling, including TCS, drop attacks (epileptic falls), and seizure clusters [10, 11, 14, 15]. It aims to provide a clearer understanding of the VNS efficacy and to identify specific seizure targets, ultimately improving the selection of suitable candidates for this treatment.

## Methods

This is a monocentric retrospective observational study, conducted within the Epilepsy Program at the IRCCS Istituto delle Scienze Neurologiche of Bologna.

All adults (> 18 years) with DRE, who were ineligible for or unresponsive to resective surgery and received VNS therapy, followed at our Center from January 2007 to December 2023 were screened. Eligibility required a minimum follow-up period of 12 months after VNS implantation.

In our cohort, the implanted VNS devices included the Model 102, Model 103 (Demipulse), Model 106 (AspireSR), and Model 1000 (SenTiva) (LivaNova, Houston, TX, USA). Most patients were implanted at IRCCS Istituto delle Scienze Neurologiche of Bologna. The surgical technique consisted of a dual-incision approach: a horizontal cervical incision on the left side of the neck for electrode placement around the vagus nerve, and an oblique lateral chest incision at the pectoral level for generator implantation. The generator was positioned subcutaneously, facilitating both wireless device programming and battery replacement procedures. All VNS devices were positioned on the left side.

Clinical data were obtained through a review of medical records and outpatient clinic visits conducted by the same neurologist at three key time points: prior to implantation (T0), 12 months post-implantation (T1) and the most recent follow-up (T2), in line with routine clinical practice. At baseline (T0), demographic data (age, sex), epilepsy etiology and duration, age at VNS implantation, and presence of encephalopathy were assessed. Information on seizure type, frequency, severity/duration was collected at each time point, while VNS-related side effects were specifically evaluated during follow-up visits. Seizure types, epilepsy etiologies, and syndromes were classified according to the ILAE classification of epilepsies [16] and epilepsy syndromes [17–19].

For the purposes of this study, we focused specifically on TCS, including both generalized TCS and focal to bilateral TCS. Drop attacks and seizure clusters were also analyzed as distinct seizure outcomes. Drop attacks consist of sudden falls resulting from either a collapse of postural muscle tone or abnormal muscle contractions in the legs, observed during atonic and tonic seizures, respectively [20, 21]. Seizure clusters were described in our study as closely grouped seizures occurring over a period ranging from minutes to two days, representing an increase in seizure frequency compared to baseline [22].

The total seizure frequency was estimated as the average over the previous 6–12 months and categorized into five levels: multidaily (I), daily (II), >1/week (III), > 1/month (IV), > 1/year (V), similarly to the outcome measures developed by the American Academy of Neurology (AAN) for standardized epilepsy care in outpatient settings [23]. The distinction between multidaily and daily seizures was made based on their differing impact reported subjectively by people with epilepsy (PWE) and/or caregivers. Seizure duration/severity was assessed through PWE and/or caregivers’ reports obtained during anamnestic reconstruction and classified into two levels: reduced or not reduced.

Seizure frequency reduction for specific seizure types—including TCS (both generalized and focal to bilateral TCS), drop attacks (atonic and tonic seizures), and seizure clusters—was determined based on reports from PWE and/or caregivers. These were categorized into three levels: seizure freedom (no seizures in the previous 6–12 months), frequency decrease, no benefit. Seizure cluster duration was similarly assessed and classified into two levels: decreased or unchanged.

The main outcomes were (1) total seizure frequency decrease at T1 and T2; (2) reduction in total seizures duration/severity at T1 and T2; (3) reduction in TCS and drop attacks frequency at T1 and T2; (4) reduction in seizure cluster frequency, duration, or both at T1 and T2. We adopted a composite outcome termed “clear benefit” to define a decrease in frequency and/or duration of clusters at each time point. Secondary outcomes included the PWE’s or caregiver’s decision to replace the battery upon or near depletion, which was considered a proxy for effectiveness, as well as assessments of safety and tolerability.

Demographic and clinical data of enrolled subjects were collected in a structured database, pseudo-anonymized to ensure participant confidentiality.

### Ethical committee

The retrospective study was conducted with the approval of the Local Ethics Committee (CE24142).

### Statistical analysis

To compute the reduction in total seizure frequency, the total seizure frequency at T1 and T2 was subtracted from the total seizure frequency at T0, resulting in two timepoint measures of category shift: one for T1 (T1-T0) and one for T2 (T2-T0).

For the specific seizure types, the proportions of seizure freedom, frequency decrease, and no benefit were calculated at T1 and T2 (as summarized in Table 3). Subjects were classified as responders if they achieved a frequency reduction or disappearance for TCS and drop attacks, or a clear benefit or disappearance for seizure clusters. Subjects who exhibited no improvement were classified as non-responders. To evaluate whether the proportions of responders were significantly different from that of non-responders (i.e., against a null hypothesis of equal proportions), a chi square test (χ^2^-test) was used. Separate analyses were conducted for T1 and T2. All the proportion tests were computed after the exclusion of “not available” (N/A) values.

## Results

### Study population

Of the 90 subjects screened, three were excluded because their follow-up period was shorter than 12 months. The final cohort consisted of 87 subjects, including 51 males, having a median age of 33 years at the time of VNS implantation. Clinical evaluations were conducted at either T1 (available in 82 cases), T2 (82 cases), or both time points (77 subjects). Table 1 summarizes the main demographic and clinical features of the study population. Three subjects had both genetic and structural etiologies, as their epileptogenic lesions were caused by pathogenic variants in the TSC1/TSC2 genes. Concomitant genetic and metabolic etiologies were identified in two individuals with Lafora disease and in one subject with GLUT1 deficiency syndrome (diagnosed after implantation). Nearly half of the individuals had a developmental and epileptic encephalopathy (DEE), including 12 cases of Lennox-Gastaut syndrome (13.8%). Three subjects had previously undergone unsuccessful resective surgery, and stereo-EEG-guided thermocoagulation had been performed in one case. The majority of subjects had high or very high seizure frequency, with more than half experiencing daily or multiple daily seizures and 75% having seizures more than weekly. Only one subject had sporadic seizures, but a substantial medication burden (6 ASMs) warranted VNS implantation.

**Table 1:** Demographics and clinical features of the study population

Study population (*n* = 87)	
Sex, *n* (%)	M 51 (58.6%); F 36 (41.4%)
Age at seizure onset (years)	
Mean ± SD (range); median	9.7 ± 8.9; 8
Age at VNS implantation (years)	
Mean ± SD (range); median	32.9 ± 13.8 (7–65); 33
Epilepsy duration prior to VNS implantation (years)	
Mean ± SD (range); median	23.7±12.8 (1–57); 23
Follow-up duration at T2 (months)	
Mean ± SD (range)	94.5 ± 59.4 (12–318)
Follow-up duration between T1 and T2 (months)	
Mean ± SD (range)	83.4 ± 58.6 (0–306)
Etiology, *n* (%)	
Genetic	10 (11.5%)
Structural	43 (49.4%)
Genetic and structural	3 (3.4%)
Genetic and metabolic	3 (3.4%)
Unknown	28 (32.2%)
Syndrome, n (%)	
Focal syndromes	24 (27.6%)
♢ Mesial Temporal Lobe Epilepsy with Hippocampal Sclerosis	4 (4.6%)
♢ Sleep Hypermotor Epilepsy (SHE)	1 (1.1%)
♢ Others	19 (21.8%)
Developmental and epileptic encephalopathy (DEE)	36 (41.3%)
♢ Lennox-Gastaut Syndrome	12 (13.8%)
♢ Dravet Syndrome	2 (2.3%)
♢ GLUT1 Deficiency Syndrome	1 (1.1%)
♢ Progressive Myoclonic Epilepsies	3 (3.4%)
♢ Others	18 (20.7%)
Unknown	27 (31%)
Seizure types across clinical history, n (%)	
Focal seizures	80 (92%)
Tonic-clonic seizures	54 (62.1%)
Drop attacks	62 (71.3%)
Seizure clusters across clinical history, n (%)	65 (74.7%)
Total seizure frequency at VNS implantation, n (%)	
multidaily	40 (46%)
daily	10 (11.5%)
> 1/week	15 (17.2%)
> 1/month	21 (24.1%)
> 1/year	1 (1.1 %)

*F* female, *M* male, *SD*standard deviation, Tonic-clonic seizures (TCS) include generalized TCS and focal to bilateral TCS

### Clinical outcomes

#### Impact on overall seizures

Clinical outcomes of VNS treatment regarding total seizures are summarized in Table 2. At T1, 37 subjects (42.5%) reported a reduction in seizure frequency of at least one category, increasing to 47 (54%) at T2. Among the 42 cases of battery depletion, 36 elected to undergo battery replacement. Five subjects did not proceed with replacement due to electrode rupture (1 case) or lack of efficacy (4 cases). Additionally, one individual was diagnosed with GLUT1 Deficiency Syndrome after implantation and discontinued VNS therapy following initiation of disease-specific treatment.

**Table 2:** Clinical outcomes: impact on overall seizures

Outcomes		T1	T2
Total seizures			
Frequency	No reduction	44 (50.6%)	34 (39.1%)
	Reduction		
	By 1 category	20 (23%)	21 (24.1%)
	By 2 categories	8 (9.2%)	18 (20.7%)
	By 3 categories	9 (10.3%)	7 (8%)
	By 4 categories	0	1 (1.2%)
	N/A	6 (6.9%)	6 (6.9%)
	Sum: ³ 1 category	37 (42.5%)	47 (54%)
Severity - duration	Reduction	59 (67.8%)	62 (71.3%)
Frequency and/or severity - duration	Reduction	65 (74.7%)	70 (80.5%)
Battery replacement	36/42 (85.7%) of cases of battery depletion		
Switching off	6 (6.9%)Insufficient benefits: 3 (3.4%)Not replaced after electrode rupture*: 2 (2.3%)N/A: 1 (1.1%)		

*N/A* not available, T1 (12 months); T2 (last follow-up); * the surgical risks were considered unacceptable

The stimulation intensity was titrated up to 1.75 mA in 80% of individuals, ranging from 1.50 mA to 2.25 mA. In three subjects, intensity was maintained below 1.75 mA either due to side effects at higher levels (2 cases) or because adequate seizure control had already been achieved at 1.50 mA (1 case). The stimulation cycle varied between 30 s on-5 min off and 30 s on-1.8 min off. The magnet was used by PWE or their caregivers in 30 cases (34.5%), demonstrating efficacy in 24 of them (80%). Factors contributing to its underuse included having only seizures that would not benefit from magnet stimulation (i.e.: brief, isolated, without aura or long post-ictal phase), or evidence of inefficacy. The auto-stimulation mode was activated in 29 cases (33.3%). 

#### Safety and tolerability

Side effects were reported in nearly half of the study population (43 cases; 49.4%), with approximately 90% of these (38 cases) being transient. Thirty-six individuals experienced voice alteration or hoarseness, with most of them (34) achieving substantial recovery within the first year. Vocal cord palsy occurred in two cases, one of which was transient. Two subjects reported a worsening of obstructive sleep apnea, which was successfully managed in one case through the day/night programming feature of the most recent device model. Electrode rupture occurred in two cases.

Two surgical complications were reported: one case of skin dehiscence and one case of superficial wound infection. Both complications were resolved with medical treatment and did not require device removal.

#### Impact on specific seizure types

Focusing on the most disabling seizure types, our findings are summarized in Table 3. A reduction in the frequency of TCS was observed in 52.9% of cases at T2. Approximately 18% and 21% of individuals achieved complete freedom from TCS at T1 and T2, respectively. Among the 55 subjects experiencing drop attacks, 12 (21.8%) experienced a disappearance of these episodes at T2.

**Table 3:** Clinical outcomes: impact on specific seizure types

Cohort	Outcome	T1	T2
Tonic-clonic seizures			
[34 at VNS implant]	Seizure freedom	6 (17.6%)	7 (20.6%)
	Reduction	15 (44.1%)	18 (52.9%)
	Unchanged	7 (20.6%)	6 (17.6%)
	N/A	6 (17.6%)	3 (8.8%)
Drop attacks			
[55 at VNS implant]	Disappeared	4 (7.3%)	12 (21.8%)
	Reduced	29 (52.7%)	24 (43.6%)
	Unchanged	11 (20%)	10 (18.2%)
	N/A	11 (20%)	9 (16.4%)
Seizure clusters			
[62 at VNS implant]	Disappeared	1 (1.6%)	4 (6.4%)
	Reduced		
	a) in frequency	10 (16.1%)	10 (16.1%)
	b) in duration	6 (9.7%)	5 (8.1%)
	c) in frequency and duration	22 (35.5%)	26 (41.9%)
	d) in frequency and/or duration	38 (61.3%)	41 (66.1%)
	Unchanged	7 (11.3%)	9 (14.5%)
	N/A	16 (25.8%)	8 (12.9%)

*N/A* not available, T1 (12 months); T2 (last follow-up)

**Fig. 1:**
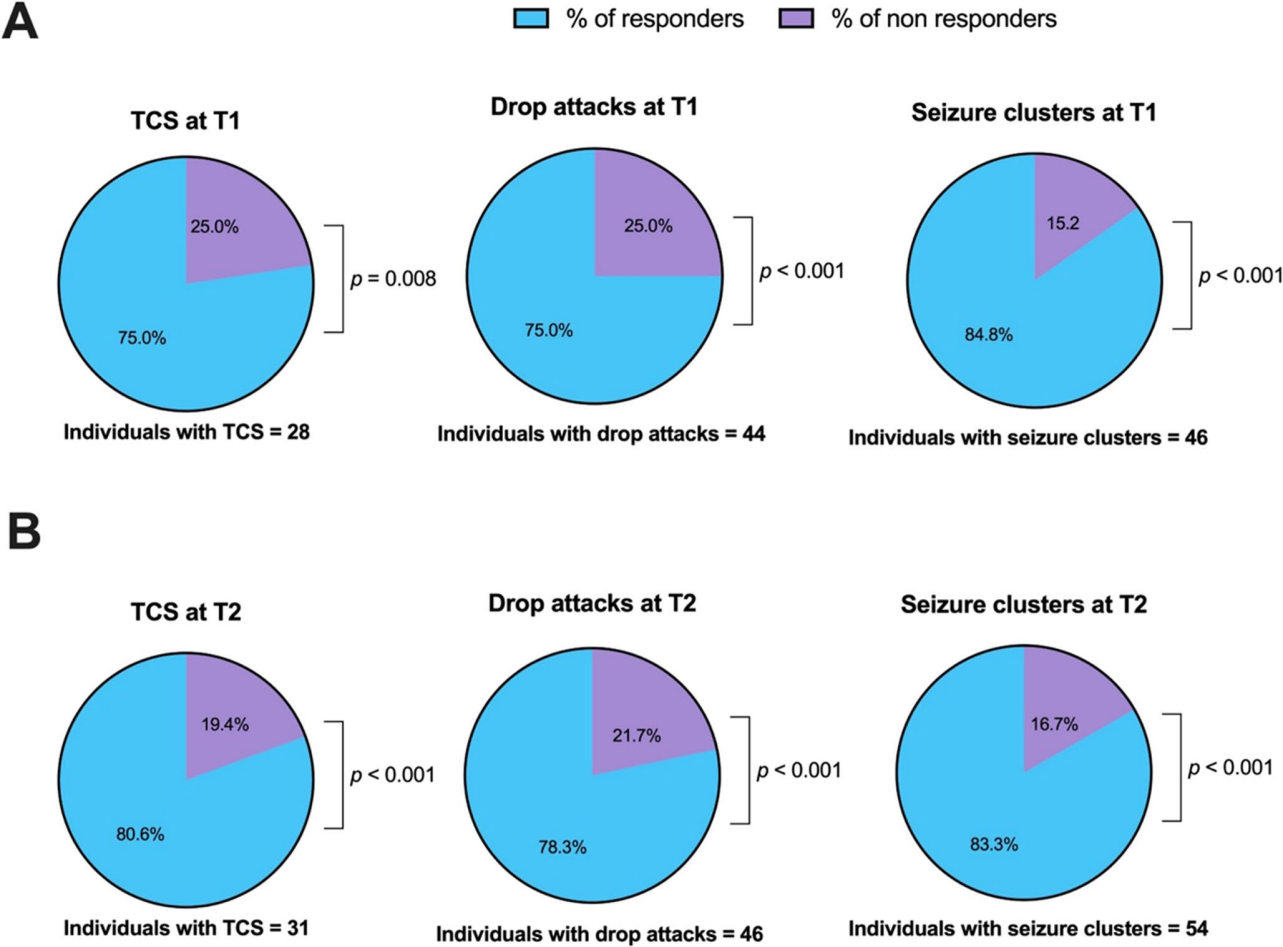
Pie charts illustrating the proportions of responders and non-responders for the specific seizure types under investigation. Responders are defined as individuals who present a disappearance or decreased frequency for TCS/drop attacks and a disappearance or reduction in frequency and/or duration for seizure clusters. Panel A refers to the percentages at T1 (12-month follow-up), while Panel B indicates those at T2 (last follow-up) among individuals presenting with TCS, drop attacks, and seizure clusters at the time of implantation. P-values from the proportion tests are provided. All the proportion tests were computed after excluding N/A values

Proportion tests comparing responders and non-responders for specific seizure types are presented in **Figure** 1. The proportion of individuals experiencing a meaningful improvement in seizure control was significantly higher than that of non-responders. Regarding TCS frequency, response rates reached 75% at T1 (χ^2^_(1)_ = 7.00, *p* = 0.008) and 80.6% at T2 (χ^2^_(1)_ = 11.6, *p* < 0.001). Similarly, among subjects with drop attacks, 75% and 78.3% showed a significant decrease in frequency at T1 (χ^2^_(1)_ = 11.0, *p* = 0.001) and at T2 (χ^2^_(1)_ = 14.7, *p* < 0.001), respectively. Lastly, among individuals presenting with seizure clusters at the time of VNS implantation, over 80% had a clear benefit at both T1 (χ^2^_(1)_ = 22.3, *p* < 0.001) and T2 (χ^2^_(1)_ = 24.0, *p* < 0.001).

## Discussion

This retrospective, single-center study involving 87 individuals with DRE treated with VNS demonstrates a long-term decrease in seizure frequency by at least one category in approximately 50% of subjects, consistent with response rates reported in the existing literature [4]. The most clinically relevant finding of our study is the marked effect of VNS on the most disabling seizure types, which surpasses its impact on overall seizure burden, thereby confirming and potentially extending previous observations with long-term real-world data. Specifically, over 70% of individuals experienced a reduction in the frequency of TCS and drop attacks, while over 80% of those with seizure clusters showed a reduction in either their frequency or duration.

A notable strength of our work lies in its extended follow-up period, with a mean duration of nearly 8 years and a maximum of 26.5 years at the last follow-up. Fewer than 10 studies to date reported follow-ups exceeding 4 years [24]. We observed comparable effectiveness at 12 months and at the last follow-up for the specific seizure types under investigation, suggesting that most of the therapeutic benefit emerges within the first-year post-implantation and remains stable over time.

Seizure frequency alone poorly represents the burden of epilepsy in people treated with VNS, as already noted by McHugh at al. [25]. In our cohort, approximately 70% of subjects reported a reduction of seizure severity or duration. This finding could be valued considering the evolving field of the patient-reported outcome measures (PROMs) [26]. By incorporating subjective measures on health outcomes, such as seizure severity, duration, and intensity, PROMs integrate the patients perspective into healthcare evaluation [26]. Similarly, a multi-modal assessment of VNS outcomes was recently performed through the administration of three validated seizure severity scales [27]. However, the retrospective design of this study prevented us from utilizing specific evaluation tools.

Interestingly, in our study, battery replacement was performed in a larger population (85.7%) of subjects than those experiencing benefits in either seizure frequency or intensity. This trend has also emerged in previous research, where tangible improvements in quality of life have motivated the continuation of VNS therapy, even in the absence of significant reductions in seizure count [24]. Specifically, VNS has demonstrated improvements in alertness, cognition and behavior [6, 28]. Several concurrent effects of VNS, such as fewer falls, shorter post-ictal periods, decreased seizure intensity, and reduced reliance on ASM, have been highlighted as equally important determinants of overall well-being [24, 29]. From these observations, a complexity within the implanted population arises that has been overlooked, thereby leading to an undervaluation of VNS broader impact [1, 7, 24, 27, 29–34].

Building on these insights, we weighted seizure frequency reduction based on seizure types to achieve a more comprehensive evaluation of VNS efficacy.

Among PWE with TCS at the time of implantation, over 70% experienced a frequency reduction at one year and last follow-up, with up to 20.6% achieving seizure freedom. These results may have implications for clinical practice, especially when considered in the context of the progressive broadening of VNS indications from focal to generalized epilepsies. Initially, VNS was indicated in focal epilepsies based on adult randomized trials that focused on this specific epilepsy type [35]. Focal epilepsies remain more commonly treated with VNS, likely due to their greater prevalence and higher potential for drug resistance [4, 36]. The detection of diffuse EEG abnormalities and generalized seizures in pediatrics cohorts treated with VNS progressively broadened its indications to generalized epilepsy [4]. A randomized controlled trial involving both focal and generalized epilepsy patients have shown no significant differences in responder rates [35]. Notably, other studies reported higher responsiveness and greater likelihood of seizure freedom in generalized or mixed seizure compared to focal seizures [4, 37–39]. More recently, the potentiality of neuromodulatory interventions has been emerging also for drug-resistant idiopathic generalized epilepsy [7, 40–42].

A study examining four seizure types – generalized TC, focal, myoclonic, and atonic - found that generalized TC and atonic seizures responded more favorably to VNS compared to the others [6]. Similarly, while evaluating the differential impact of VNS on major versus minor seizures – classified based on the degree of motor phenomena and awareness impairment - a notably higher efficacy was detected among PWE with major seizures, such as TCS, with a response rate exceeding 80% [30]. Recent research indicate that approximately half of patients with pre-implantation TCS experienced prolonged periods of TCS freedom with VNS, with responder rates of 78.2% and a median seizure frequency reduction of 96.5% at 36 months [43].

The results from our cohort support the high efficacy of VNS in alleviating these seizures, the most debilitating and hazardous type. Extended TCS remission is associated with improved physical and psychosocial functioning, reduced SUDEP risk, and fewer injury-related incidents [43]. PWE experiencing significant impairments due to intractable TCS may represent promising candidates for VNS therapy. While full seizure control following adjunctive VNS therapy is uncommon, durable remission from TCS seizures is achievable and has a transformative effect on patients’ quality of life. In line with findings from the Comprehensive Outcomes Registry in Subjects with Epilepsy Treated with Vagus Nerve Stimulation Therapy^TM^ (CORE-VNS), which recently demonstrated a frequency reduction of generalized TCS in over 70% of DRE people following 12 months of VNS [44], our results lend support to the early consideration of non-pharmacological adjunctive therapies in individuals who continue to experience TCS despite appropriate trials of two anti-seizure medications [45].

Falling episodes have been described in association of focal seizures, presenting with myoclonic, tonic, tonic-postural, atonic semiology [46]. Previous studies have demonstrated VNS efficacy in managing generalized seizures associated with Lennox Gastaut Syndrome (LGS) [7], including atonic seizures. When the sudden loss of muscle tone affects all muscle groups, the resulting unpredictable falls often lead to severe and repeated trauma. Atonic seizures carry a very poor prognosis, as almost all patients exhibit resistance to multiple ASM. Corpus callosotomy (CC) has been a mainstay treatment due to its proven efficacy in managing atonic seizures. The introduction of VNS has also shown promise in reducing the frequency of drop attacks, albeit to a lesser extent than CC [47]. Given the irreversible nature and potential complications of CC, VNS is often considered a preferred first-line surgical option. For PWE with limited treatment alternatives, any improvement in seizure frequency, however modest, may be deemed clinically significant [35]. In our cohort, over 70% of subjects experiencing drop attacks, regardless of etiology or specific semiology, showed a reduction or complete resolution of these episodes, supporting the significant utility of VNS therapy in such cases.

In our study population, more than 80% of subjects with seizure clusters presented a reduction in either the duration, frequency, or both. To our knowledge, no previous study has thoroughly examined the specific effects of VNS on seizure clusters. However, in LGS, VNS has been linked to a significant decrease in both the occurrence and recurrence of status epilepticus (SE) [48]. Level IV evidence from case reports indicates that acute VNS implantation can interrupt refractory and super-refractory status epilepticus in 74% of patients [12]. Following VNS therapy, clinicians described improvements in seizure clustering and the post-ictal state [28] in both adults and children [4, 34]. Therefore, even when reductions in overall seizure frequency are modest, PWE may experience substantial improvements in quality of life due to less severe seizures and fewer episodes requiring rescue therapy or even, as in the cases of full-blown SE, hospital admissions [48].

We propose that the effectiveness of VNS should not be evaluated solely by its ability to lower overall seizure frequency, as in curative surgery, but rather by its capacity to mitigate the most severe seizures types, which can significantly improve daily functioning. Our findings may also open new avenues for research into the underlying mechanisms of VNS therapy. Given the clinical evidence supporting its role in controlling TCS, drop attacks, and seizure clusters, it is worth investigating whether neuromodulation directly targets the pathophysiological substrates of these manifestations – all of which involve disrupted synchronization dynamics [22, 40, 49, 50].

### Limitations

This study has some limitations that should be acknowledged. The reduction in both total and specific seizure types was not based on an exact seizure count or formal classification system, introducing potential bias and lowering the precision of the reported outcomes. However, a structured interview was conducted at each time-point by the same neurologist, ensuring reliability in data collection. Moreover, the adoption of seizure frequency categories enabled us to address the practical challenges associated with accurately enumerating seizures in patients with DRE, particularly those experiencing multiple seizures per day, which constituted nearly half of our cohort at the implantation time. PWE frequently encounter difficulties in providing precise seizure counts due to impaired awareness during seizures, lack of witnesses, or misinterpretation of paroxysmal events. Alternative outcome measures, such as the number of seizure-free days, have been suggested to better capture improvements in daily functioning. By using broad frequency bands, the study design allowed for more feasible and reliable patient reporting and data collection through consultation of medical records [23, 27].

Furthermore, while total seizure frequency was computed as a measure of category shift from T0 to T1 and T2, no shift category could have been computed for the specific seizure types. Their presence was assessed at T0, and any reduction or disappearance was inferred from the PWE and/or caregivers’ reported evaluation at T1 and T2. Nevertheless, the seizure estimations based on outpatient visits reports align with those of most retrospective studies on this topic (e.g [4, 7]).,. Given the qualitative nature of the data and the absence of a control group, this study warrants future investigation to quantify the augmentation effects of VNS therapy on specific seizure types.

## Conclusion

Identifying individuals who are likely to respond to VNS prior to implantation could optimize candidate selection, reduce unnecessary VNS surgical procedures and their related morbidity, and lessen the economic burden of treatment. Our findings underscore the contribution of VNS in reducing the impact of epilepsy, particularly through its effectiveness in targeting the most disabling seizure types. From a clinical standpoint, early identification of drug resistance and timely consideration of VNS should be encouraged in PWE, especially in those presenting with refractory tonic–clonic seizures, drop attacks, or seizure clusters who are not candidates for curative surgery.
